# Uveal Effusion Following Yttrium Aluminum Garnet (YAG) Iridotomy in a Patient on Acetazolamide: A Case Report

**DOI:** 10.7759/cureus.108298

**Published:** 2026-05-05

**Authors:** Ibrahim El Amrani, Marc Schrooyen, Majedeline Rahali, Elie Motulsky

**Affiliations:** 1 Department of Ophthalmology, Erasme Hospital, Université Libre de Bruxelles (ULB), Brussels, BEL; 2 Department of Ophthalmology, Saint-Pierre Hospital, Université Libre de Bruxelles (ULB), Brussels, BEL

**Keywords:** acetazolamide, choroidal detachment, ocular hypertension, pilocarpine, prophylaxis, uveal effusion, yag laser iridotomy

## Abstract

To report a case of uveal effusion after yttrium aluminum garnet (YAG) laser iridotomy in a hyperopic patient on acetazolamide, illustrating the possible interaction between post-laser inflammation and medication-induced changes in intraocular fluid dynamics.

A 73-year-old patient was referred for left ocular hypertension and was found to have bilaterally narrow iridocorneal angles, confirmed by gonioscopy and anterior segment optical coherence tomography (AS-OCT). After initial treatment with oral acetazolamide and a prostaglandin analog eye drop, a YAG laser peripheral iridotomy was performed in the left eye following pilocarpine instillation. Six days later, the patient developed persistent peripheral blurred vision in the treated eye. Fundus examination revealed an extensive peripheral serous choroidal detachment with a peripapillary serous retinal detachment. Discontinuation of acetazolamide and initiation of intensive topical corticosteroids led to a progressive resolution of the effusion within one month. Given the risk of recurrence in predisposed eyes and the absence of acute symptoms in the fellow eye, prophylactic iridotomy was deferred in favor of close monitoring.

This case illustrates the potential role of an interaction between post-laser inflammation and pharmacologically induced transscleral pressure changes in the occurrence of uveal effusion. By reporting this case, we aim to raise awareness of this rare complication among clinicians to promote early diagnosis and prevent avoidable therapeutic complications.

## Introduction

Uveal effusion refers to the abnormal accumulation of serous fluid in the suprachoroidal space, leading to choroidal elevation and detachment of the choroid and retina. This condition may occur idiopathically (as in uveal effusion syndrome) or secondary to various triggers such as intraocular surgery, trauma, malignancy, medications, or inflammation [1]. Yttrium aluminum garnet (YAG) laser peripheral iridotomy (LPI), although a gold standard for preventing angle-closure glaucoma, can induce intraocular inflammation and transient changes in intraocular pressure, and has been reported to precipitate ciliochoroidal effusions in susceptible eyes [2]. In addition, certain medications - notably sulfonamide derivatives like acetazolamide - have been implicated in causing choroidal detachment through distinct non-inflammatory mechanisms involving ciliochoroidal effusion [3]. We report a case of multifactorial uveal effusion following YAG iridotomy in a hyperopic patient on acetazolamide. This case highlights the interplay between post-laser inflammation and drug-induced alterations in intraocular fluid dynamics.

## Case presentation

**Figure 1:**
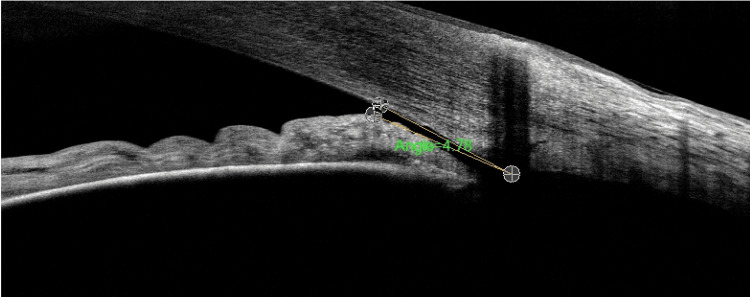
Anterior segment optical coherence tomography (AS-OCT) temporal radial scan showing a narrow iridocorneal angle in the left eye.

A 73-year-old man with a history of hypercholesterolemia and asthma was referred by his optician to our clinics for left ocular hypertension detected during a routine examination. The initial ophthalmologic examination revealed bilateral high hyperopia, with refraction of +7.25 D (−2.00 D × 70°) in the right eye and +5.50 D (−1.25 D × 123°) in the left eye. Best-corrected visual acuity was 20/20 in both eyes. Goldmann applanation tonometry showed an intraocular pressure (IOP) of 14 mmHg in the right eye and 28 mmHg in the left eye. Slit-lamp examination and gonioscopy revealed a shallow anterior chamber with a closed angle in both eyes. Anterior segment optical coherence tomography (AS-OCT) of the left eye corroborated these findings, confirming a very narrow angle of 4.78° (Figure 1). Fundus examination was unremarkable. 

Treatment was initiated with oral acetazolamide 250 mg twice daily and a prostaglandin analogue (travoprost 0.004%) once daily in the evening. Four days later, an Nd:YAG laser iridotomy was performed on the left eye following the instillation of pilocarpine 1%. It is noteworthy that shortly after pilocarpine instillation, the patient reported the onset of transient headaches.

**Figure 2:**
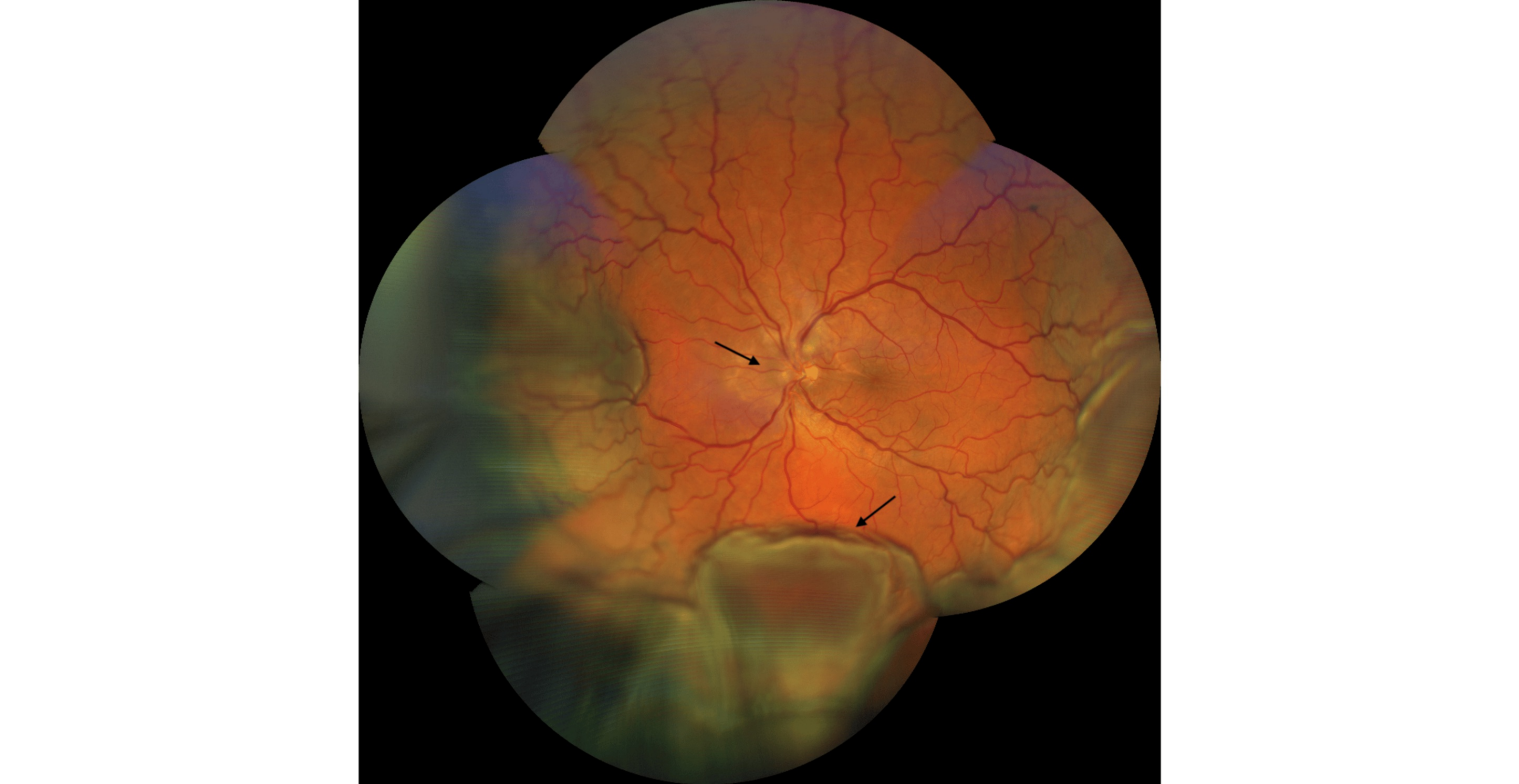
Wide-field fundus photograph (CLARUS 500), showing an extensive peripheral choroidal detachment with peripapillary serous retinal detachment and macular sparing (arrows) in the left eye. CLARUS 500, Carl Zeiss Meditec AG, Jena, Germany

Six days after the iridotomy, the patient presented with blurred peripheral vision in the left eye. Visual acuity remained 20/20 in both eyes, and IOP was 15 mmHg bilaterally. Slit-lamp examination revealed conjunctival hyperemia in the left eye, and fundus examination showed a broad serous choroidal detachment involving the inferior, nasal, and temporal quadrants, associated with a peripapillary serous retinal detachment and retinal folds (Figure 2).

**Figure 3:**
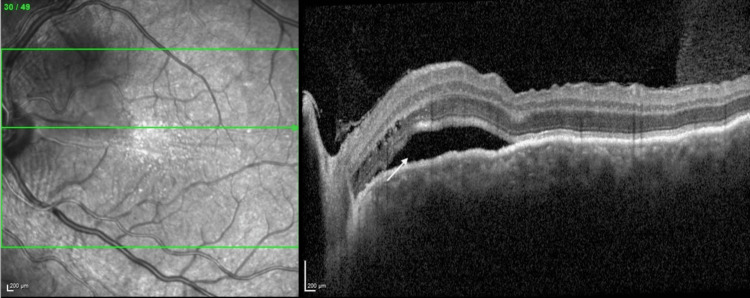
Optical coherence tomography images showing a peripapillary serous retinal detachment (arrow) with retinal folds in the left eye.

**Figure 4:**
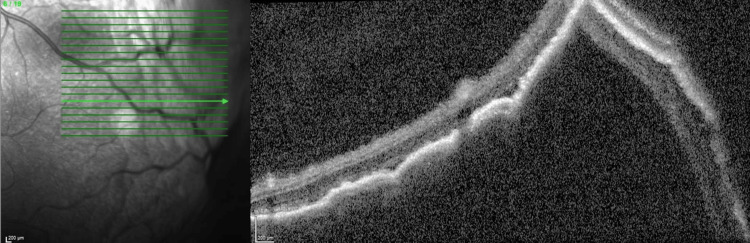
Optical coherence tomography images showing a peripheral serous choroidal detachment in the left eye.

Ocular biometry revealed axial lengths of 20.98 mm in the left eye and 21.00 mm in the right eye, consistent with high hyperopia and anatomically short eyes. Macular and peripheral OCT confirmed these findings (Figures 3, 4), additionally revealing a subfoveal choroidal thickness of approximately 330 µm, indicating mild choroidal congestion. No vitritis or retinal tear was observed.

Given the clinical presentation, a diagnosis of post-iridotomy uveal effusion was made, potentially exacerbated by the acetazolamide-pilocarpine combination. Medical management included discontinuation of both acetazolamide and the prostaglandin analogue, with initiation of topical prednisolone acetate 1% prescribed every hour while awake, followed by gradual tapering over one month.

**Figure 5:**
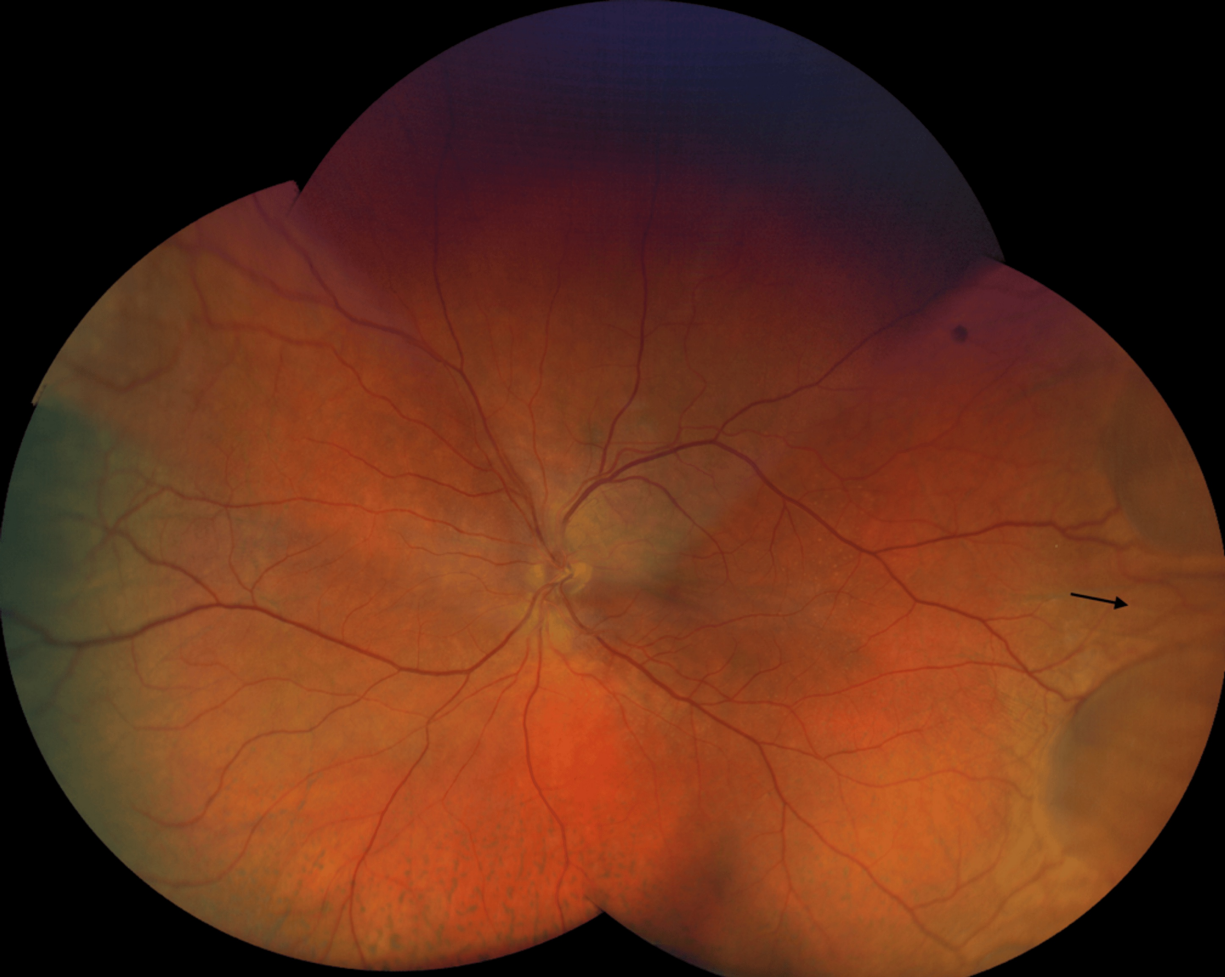
Wide-field fundus photograph (CLARUS 500) showing a residual temporal choroidal detachment (arrow) in the left eye. CLARUS 500, Carl Zeiss Meditec AG, Jena, Germany

One month after the laser, the patient reported nearly complete resolution of his peripheral visual blurring, with visual acuity maintained at 20/20. The IOP remained stable at 16 mmHg in the right eye and 15 mmHg in the left eye. The left iridotomy remained patent, with no signs of anterior chamber inflammation. Fundus examination showed progressive resorption of the choroidal detachment, with a residual temporal detachment (Figure 5). In accordance with the patient’s wishes, no prophylactic iridotomy was performed on the right eye, and regular follow-ups were arranged.

## Discussion

Uveal effusion is a rare complication of certain ocular procedures and medications, especially in eyes with predisposing anatomy. Hyperopic or nanophthalmic eyes feature a crowded anterior segment and often a thickened sclera and choroid that impede transscleral fluid outflow. This leads to choroidal congestion and a propensity for effusion even after minor triggers. In our hyperopic patient, the subfoveal choroidal thickness of approximately 330 µm-measured after LPI, may reflect either preexisting choroidal congestion or secondary changes induced by the effusion, both of which likely contribute to the observed susceptibility [1].

In the present case, a sequence of iatrogenic factors contributed to the unilateral effusion. The first insult was pharmacological: acetazolamide, a sulfonamide carbonic anhydrase inhibitor, can induce bilateral ciliochoroidal effusions leading to secondary angle closure. The proposed mechanism involves drug-induced breakdown of the ciliary body barrier and osmotic fluid shifts into the suprachoroidal space [3]. Krieg & Schipper’s “eicosanoid” theory further posits that such drug-induced ciliary body edema is mediated by inflammatory mediators: prostaglandins cause increased vascular permeability and fluid leakage, while leukotrienes induce ciliary muscle spasm and anterior movement of the lens-iris diaphragm [4].

Pilocarpine, administered prior to laser iridotomy, likely exacerbated the situation. Although miotics help relieve pupillary block, pilocarpine can paradoxically worsen angle closure when ciliary edema is present. Contracting the ciliary muscle and loosening the zonules, it allows the lens to thicken and shift forward, pushing the iris into an already narrow angle. Indeed, our patient’s headache shortly after pilocarpine instillation suggests significant ciliary muscle spasm or anterior displacement of the lens-iris diaphragm, lending clinical support to this proposed mechanism. In a scenario of incipient uveal effusion, this forward lens-iris diaphragm shift can further raise posterior segment pressure and crowd the angle, compounding the effusive process [5].

The LPI was the final trigger that made the effusion clinically apparent. Laser photodisruption initiates an intraocular inflammatory cascade; even uncomplicated LPI can acutely elevate prostaglandin and cytokine levels (such as IL-6, IL-1β, and TNF-α) in the aqueous humor [6].

These mediators increase choroidal vascular permeability and could exacerbate any preexisting choroidal congestion. While this post-laser inflammatory response is usually mild, in a predisposed eye with compromised uveoscleral outflow, it may suffice to tip the balance into a frank effusion. Case reports have documented recurrent uveal effusions after LPI attributed to laser-induced inflammation and choroidal fluid transudation; even incomplete iridotomies have been associated with exudative retinal detachment, underscoring that intense localized inflammation can precipitate this complication [2-7].

In our patient, the LPI in the left eye likely served as the final trigger, compounding the effects of acetazolamide and pilocarpine to produce the extensive choroidal detachment. Notably, only the treated eye developed an effusion, whereas the fellow eye - exposed to the same systemic medications - remained effusion-free, highlighting the pivotal role of the local laser-induced inflammation.

Management of uveal effusion in this context centers on reversing the inciting factors and controlling inflammation. In our case, we promptly discontinued acetazolamide and initiated intensive topical corticosteroids.

This conservative approach led to a gradual resolution of the choroidal detachment over one month, with the retina reattaching spontaneously. Similar outcomes have been reported in other cases of drug-induced or post-laser effusions, where removal of the offending drug and medical therapy resulted in complete recovery, avoiding the need for surgery [8]. It is worth noting that in truly idiopathic uveal effusion syndrome (where no removable precipitant exists), surgical measures are often required; procedures such as partial-thickness sclerectomies to create scleral windows have achieved anatomical reattachment in the majority of such eyes [9].

Given this predisposition, any future intraocular procedure - such as cataract extraction - should warrant special precautions; specifically, prophylactic partial-thickness sclerectomies in all four quadrants at the time of lens surgery have been recommended to facilitate transscleral drainage, relieve suprachoroidal fluid accumulation, and thereby reduce the risk of another uveal effusion [1].

An important clinical question in this scenario is how to manage the fellow eye, given its narrow-angle anatomy. Prophylactic iridotomy would ordinarily be advised to prevent acute angle closure. However, recent evidence suggests that many anatomically narrow angles do not progress to symptomatic angle closure or glaucoma if simply observed. The Zhongshan Angle-Closure Prevention (ZAP) trial (and the Asymptomatic Narrow Angle-Laser Iridotomy Study, ANA-LIS) reported that the annual risk of progression from an asymptomatic narrow angle to acute angle closure or elevated IOP is well under 1% without laser treatment [10-12]. These findings have prompted a more individualized approach to prophylaxis.

In our patient, the potential benefit of a preventive LPI in the fellow eye needed to be weighed against the demonstrated risk of inducing a uveal effusion. There is reason to be cautious: the left eye’s response suggests an idiosyncratic predisposition to choroidal effusion with laser, and others have noted that certain individuals may be inherently prone to recurrent effusions after ocular procedures [2]. Considering the low spontaneous progression risk and the patient’s history, we elected to forego an immediate iridotomy in the right eye. Instead, the patient was placed under close surveillance with periodic gonioscopy and intraocular pressure checks. This strategy avoids exposing the fellow eye to another potential effusive insult while ensuring that any angle-closure progression will be detected and managed promptly.

## Conclusions

This case illustrates how common glaucoma interventions can unexpectedly interact to produce uveal effusion and serous retinal detachment in a susceptible eye. Early recognition of this rare complication was crucial: prompt drug cessation and aggressive anti-inflammatory treatment achieved complete resolution without invasive procedures. Awareness of this potential drug-laser interaction can help clinicians prevent misdiagnosis and anticipate complications in selected patients.

## References

[1] Uyama M, Taketani Y, Kozaki J (2000). Uveal effusion syndrome: clinical features, surgical treatment, histologic examination, and physiologic study. Ophthalmology.

[2] Sakai H, Yonahara M, Sakai M (2017). Recurrent uveal effusion after laser iridotomy. Case Rep Ophthalmol.

[3] Bayer A, Moroi SE (2010). Acetazolamide and bilateral uveal effusion with secondary acute angle-closure glaucoma. Glaucoma Today.

[4] Krieg PH, Schipper I (1996). Drug-induced ciliary body oedema: a new theory. Eye (Lond).

[5] Yang MC, Lin KY (2019). Drug-induced acute angle-closure glaucoma: a review. J Curr Glaucoma Pract.

[6] Er H, Doganay S, Evereklioglu C, Turkoz Y, Gündüz A, Borazan M, Ozyalin F (2002). Comparison of the effects of argon and neodymium:YAG laser iridotomy on cytokines in the rabbit aqueous humor. Eur J Ophthalmol.

[7] Iannetti L, Mastrogiuseppe E, Gnolfo E, Spagnolo S, Gharbiya M (2024). Choroidal effusion with exudative retinal detachment following non perforating YAG-laser peripheral iridotomy: a case report. Ocul Immunol Inflamm.

[8] Khanam Z, Krishnamoorthy S, Baskaran V, Mashruwala A (2024). Acetazolamide - friend to foe. Indian J Ophthalmol Case Rep.

[9] Hodges B, Omoruyi F, Allison K (2024). Uveal effusion syndrome: a case report. J Med Case Rep.

[10] He M, Jiang Y, Huang S (2019). Laser peripheral iridotomy for the prevention of angle closure: a single-centre, randomised controlled trial. Lancet.

[11] Baskaran M, Kumar RS, Friedman DS (2022). The Singapore asymptomatic narrow angles laser iridotomy study: five-year results of a randomized controlled trial. Ophthalmology.

[12] Gupta V (2024). Rethinking laser iridotomy in angle-closure disease. Indian J Ophthalmol.

